# Proteomic Analysis of miR-195 and miR-497 Replacement Reveals Potential Candidates that Increase Sensitivity to Oxaliplatin in MSI/P53wt Colorectal Cancer Cells

**DOI:** 10.3390/cells8091111

**Published:** 2019-09-19

**Authors:** Dennis Poel, Lenka N.C. Boyd, Robin Beekhof, Tim Schelfhorst, Thang V. Pham, Sander R. Piersma, Jaco C. Knol, Connie R. Jimenez, Henk M.W. Verheul, Tineke E. Buffart

**Affiliations:** 1 Department of Medical Oncology, Cancer Center Amsterdam, Amsterdam UMC, VU University Medical Center, 1081HV Amsterdam, The Netherlands; dennis.poel@radboudumc.nl (D.P.); r.beekhof@amsterdamumc.nl (R.B.); t.schelfhorst@nki.nl (T.S.); t.pham@amsterdamumc.nl (T.V.P.); s.piersma@amsterdamumc.nl (S.R.P.); j.knol@amsterdamumc.nl (J.C.K.); c.jimenez@amsterdamumc.nl (C.R.J.);; 2 Department of Medical Oncology, Radboud University medical center, 6525GA Nijmegen, The Netherlands; 3 Antoni van Leeuwenhoek, Department of Gastrointestinal Oncology, 1066CX Amsterdam, The Netherlands

**Keywords:** microRNA, chemotherapy, proteomics, colorectal cancer

## Abstract

Most patients with advanced colorectal cancer (CRC) eventually develop resistance to systemic combination therapy. miR-195-5p and miR-497-5p are downregulated in CRC tissues and associated with drug resistance. Sensitization to 5-FU, oxaliplatin, and irinotecan by transfection with miR-195-5p and miR-497-5p mimics was studied using cell viability and clonogenic assays in cell lines HCT116, RKO, DLD-1, and SW480. In addition, proteomic analysis of transfected cells was implemented to identify potential targets. Significantly altered proteins were subjected to STRING (protein-protein interaction networks) database analysis to study the potential mechanisms of drug resistance. Cell viability analysis of transfected cells revealed increased sensitivity to oxaliplatin in microsatellite instable (MSI)/P53 wild-type HCT116 and RKO cells. HCT116 transfected cells formed significantly fewer colonies when treated with oxaliplatin. In sensitized cells, proteomic analysis showed 158 and 202 proteins with significantly altered expression after transfection with miR-195-5p and miR-497-5p mimics respectively, of which CHUK and LUZP1 proved to be coinciding downregulated proteins. Resistance mechanisms of these proteins may be associated with nuclear factor kappa-B signaling and G1 cell-cycle arrest. In conclusion, miR-195-5p and miR-497-5p replacement enhanced sensitivity to oxaliplatin in treatment naïve MSI/P53 wild-type CRC cells. Proteomic analysis revealed potential miRNA targets associated with the cell-cycle which possibly bare a relation with chemotherapy sensitivity.

## 1. Introduction

Colorectal cancer (CRC) is a common health problem and one of the leading causes of cancer death worldwide [1]. Of the on average 14,000 patients diagnosed with CRC per year in the Netherlands, approximately 20% present with metastases at diagnosis and 50% will develop metastases during the course of disease. These patients are commonly treated with systemic combination therapy [2,3], often consisting of a fluoropyrimidine (5-FU or oral capecitabine) in combination with oxaliplatin or irinotecan and bevacizumab (monoclonal antibody against vascular endothelial growth factor), cetuximab or panitumumab (monoclonal antibody against epidermal growth factor receptor) [4,5]. Although patients can benefit from these (combination) treatment strategies, one of the main causes of treatment failure is resistance to chemotherapy, which eventually occurs in the majority of patients with advanced CRC (mCRC) [6].

MicroRNAs (miRNAs) have recently emerged as important players in therapy resistance in mCRC [7]. miRNAs are small non-coding RNA molecules that post-transcriptionally regulate gene expression by complementary binding to target messenger RNA (mRNA) transcripts, causing degradation of mRNA or preventing translation into protein [8]. As such, miRNAs interact with extensive intracellular signaling networks and have effect on many processes involved in cancer such as metabolic homeostasis, proliferation, and apoptosis [6]. Aberrant expression of miRNAs can contribute to carcinogenesis by excessive suppression of anti-tumor genes or a lack of suppression of oncogenes [7,9]. Furthermore, miRNAs target genes within key pathways involved in drug resistance and thus sensitivity to chemotherapeutics [6].

Non-coding RNA molecules, including miRNAs, are a class of epigenetic modifiers that are promising therapeutic targets [10]. Several studies have focused on chemotherapy sensitization by mimicking or antagonizing the expression of miRNAs. Replacement of miRNAs with mimics has been reported to improve treatment sensitivity in human CRC cell lines and recent studies demonstrated that miRNA mimics can safely be administrated in vivo in mouse models, further emphasizing miRNAs as potential candidates for enhancing treatment in clinical practice [11].

The first translation of miRNA mimic-based therapy in clinical practice was Miravirsen, an inhibitor of miR-122, used for treatment of patients with hepatitis C virus infection [12]. For solid tumors clinical trials have been started using miRNA mimic-based therapy. Treatment with miR-16 mimic loaded minicells in patients with malignant pleural mesothelioma (MPM) and advanced non-small cell lung cancer (NSCLC) resulted in anti-tumor activity with acceptable toxicity [11].

In our previous study we analyzed 220 fresh-frozen CRC tissue samples using next-generation sequencing (NGS) and identified 222 miRNAs significant differentially expressed in tumor tissue compared to normal colon mucosa [13], including 135 miRNAs with significantly higher expression and 87 miRNAs with significantly lower expression in the tumor. Among the most significantly lower expressed miRNAs in tumor tissue were miR-195-5p and miR-497-5p, both belonging to the miR-15/16/195/424/497 family, important in cell-cycle regulation by targeting signaling pathways involved in cell division, apoptosis and proliferation [14,15]. Previous studies have shown that replacement of these specific miRNAs could potentially improve sensitivity to anti-tumor therapies such as cisplatin in human epidermoid cells and radiotherapy or 5-FU treatment in CRC cells [16,17]. However, an unambiguous establishment of their involvement in resistance mechanisms against common therapies for patients with mCRC, such as oxaliplatin and irinotecan, remains to be investigated.

Considering the above, the aim of this study was to increase sensitivity to chemotherapy used for treatment of patients with mCRC by restoring expression of the downregulated miRNAs in vitro in CRC cell lines using miRNA mimics of miR-195-5p and miR-497-5p. In addition, label-free proteomics was performed to unravel potential mechanisms associated with resistance to these chemotherapies.

## 2. Materials and Methods

### 2.1. Cell Culture

The human CRC cell lines HCT116, RKO, DLD-1, and SW480 were selected for this study. Molecular characteristics are presented in Table S1. Cell lines were cultured in Dulbecco’s Modified Eagle’s high glucose with ultra-glutamine Medium (DMEM), supplemented with 20 mM Hepes buffer, 1% Penicillin/Streptomycin (Lonza BioWhittaker, Verviers, Belgium) and 10% non-heat inactivated fetal bovine serum South America (Biowest, Nuaillé, France). The cells were kept at 37 °C and 5% CO_2_ in a humidified incubator. All cell lines were authenticated and tested negative for mycoplasma contamination.

### 2.2. miRNA Transfection

Cells were seeded in a 6-well plate and cultured for 24 h to a confluency of 60–80%. Twenty-four hours after seeding, the cells were transfected with miR-195-5p (10µM) and miR-497-5p (10 µM) miRCURY LNA miRNA mimics (Exiqon, Vedbaek, Denmark) and a negative control mimic synthetic cel-miR-39-3p (Qiagen, Hilden, Germany) using Lipofectamine RNAiMAX transfection reagent (Thermo Fisher Scientific, Landsmeer, Netherlands) according to the manufacturer’s protocol.

### 2.3. Cell Viability

Twenty-four hours after transfection HCT116, RKO, DLD-1 (3000 cells per well) and SW480 (2500 cells per well) were seeded in triplicate in flat bottom 96-well plates and allowed to attach for 16 to 18 h. A separate control plate was seeded in a similar way and used as a t = 0 plate. Chemotherapeutics were prepared in solutions of 10 mM 5-fluorouracil (5-FU) (Sigma-Aldrich, St. Louis, MO, USA), 12.6 mM oxaliplatin (OHP) (Sigma-Aldrich, Zwijndrecht, Netherlands) and 200 mM irinotecan (SN-38) (Rhone-Poulenc Rorer, Lyon, France) in dimethyl sulfoxide (DMSO; Sigma-Aldrich) and stored at −20 °C. Drug dilutions of 5-FU (0–96 µM), OHP (0–12.8 µM) and SN-38 (0–200 nM) were added to each cell line. After 72 h cell viability was tested using tetrazolium 3-(4,5-dimethylthiazol-2-yl)-2,5-diphenyltetrazolium bromide (MTT) reagent (5 mg/mL) (Sigma-Aldrich) and quantified as previously described with the adjustment of using 50 µL MTT and 150 µL DMSO [18]. IC_50_ values were determined using R studio (version 1.1.423) with R software (version 3.5.0) and R-package N-Parameter Logistic Regression (NPLR) downloaded from Bioconductor. IC_50_ values of three independent experiments were compared using an ordinary one-way ANOVA.

### 2.4. Clonogenic Assay

Twenty-four hours after transfection 200 cells per cell line were seeded in duplicate in 6-well plates and allowed to attach for 24 h. Cells were treated with cell line specific IC_50_ values for each chemotherapeutic regimen. Cells transfected with cel-miR-39-3p were used as negative control. Additionally, a non-transfected cell line was included in each experiment to evaluate the effect of the transfection itself on the sensitivity to each chemotherapeutic drug. After 72 h cells were washed twice with Phosphate Buffered Saline (PBS) and incubated at 37 °C and 5% CO_2_ in a humidified incubator with drug free DMEM for 5 days (RKO) or 10 days (HCT116, DLD-1, SW480). Medium was renewed once after 5 days. Colonies were fixed with fixation solution (acetic acid/methanol 1:7), stained with crystal violet (Sigma-Aldrich) and counted according to the ‘Clonogenic assay of cells in vitro’ protocol [19]. The average number of colonies of three independent duplicate experiments were compared using an ordinary one-way ANOVA.

### 2.5. RNA Extraction

Forty-eight hours after transfection RNA was extracted using TRIzol reagent (Invitrogen, Carlsbad, CA, USA), according to the manufacturer’s protocol and stored at −80 °C until use. RNA quantity was analyzed using Nanodrop-2000 (Thermo scientific, Waltham, MA, USA). Three independent RNA isolations were performed for each cell line under four conditions: non-transfected, cel-miR-39-3p transfected, miR-195-5p mimic transfected, and miR-497-5p mimic transfected.

### 2.6. Quantification of miRNA and mRNA Expression

miRNA expression was quantified using miRCURY LNA Polymerase chain reaction (PCR) primer sets (Exiqon): cel-miR-39-3p, hsa-miR-16-5p, hsa-miR-195-5p and hsa-miR-497-5p, and analyzed as described before [20]. mRNA was quantified using 100 ng total RNA which was reverse transcribed into cDNA using the iScript™ cDNA Synthesis Kit according the manufacturer’s protocol (Bio-Rad, Mitry-Mory, France). mRNA expression was evaluated by RT-qPCR using ExiLENT SYBR^®^ Green master mix (Exiqon). Primers for WEE1 [21], CCNE1 [22], E2F3 [23], and reference gene GAPDH (reverse: 5′-CATGGTTCACACCCATGACG-3′, forward: 5′-GGGAAGCTTGTCATCAATGG-3′) were synthesized by Eurogentec (Eurogentec, Liège, Belgium). mRNA expression was evaluated by RT-qPCR using 3 µL cDNA diluted 1:40 with nuclease free H_2_O, 5 µL ExiLENT SYBR^®^ Green master mix (Exiqon), 1 µL reverse and 1 µL forward primer, using the same PCR amplification protocol as for miRNA quantification. miRNA expression levels were normalized with miR-16-5p and mRNA expression levels with GAPDH [24]. Results were compared using an ordinary one-way ANOVA.

### 2.7. Proteomic Analysis

Cell lines HCT116, RKO, DLD-1, and SW480 were plated in 6 wells plates and transfected with cel-miR-39-3p, miR-195-5p mimic, and miR-497-5p mimic. Transfections were performed in triplicate. 48 h after transfections cell lysates were collected for proteomic analysis. Cells were washed three times with PBS and collected into 50 µL lysis buffer (4× NuPage lithium dodecyl sulfate (LDS)Sample Buffer (Invitrogen), 10% DTT, H_2_O). Cell lysates were immediately heated for 10 min at 99 °C and stored at −80 °C until further use. Next, 25 µL cell lysate of each sample was loaded on self-casted 7.5% acrylamide gels and run for 1 h at 120 volts. To calculate the optimal loading volume per sample, the gels were stained and fixed using 34% methanol containing 3.5% phosphoric acid and coomassie brilliant blue G-250 (Thermo Fisher scientific, Landsmeer, The Netherlands). After calculating the optimal loading volume per sample, samples were again loaded on self-casted 7.5% acrylamide gels and run for 25 min at 100 volts. Gels were again fixed and stained for four hours using a similar coomassie brilliant blue G-250 methanol mixture and destained using milliQ water and stored at 4 °C until in-gel digestion for LC/MS-MS. Next, each sample was cut from the gel and underwent in-gel digestion followed by LC/MS-MS analysis described by Piersma et al. [25]. Briefly, peptides were separated by an Ultimate 3000 nanoLC-MS/MS system (Dionex LC-Packings, Amsterdam, The Netherlands) equipped with a 40 cm × 75 μm ID fused silica column custom packed with 1.9 μm 120 Å ReproSil Pur C18 aqua (Dr Maisch GMBH, Ammerbuch-Entringen, Germany). After injection, peptides were trapped at 6 μL/min on a 10 mm × 100 μm ID trap column packed with 5 μm 120 Å ReproSil Pur C18 aqua in 0.05% formic acid. Peptides were separated at 300 nL/min in a 10–40% gradient (buffer A: 0.5% acetic acid (Fischer Scientific), buffer B: 80% ACN, 0.5% acetic acid) in 90 min (120 min inject-to-inject). Eluting peptides were ionized at a potential of +2 kVa into a Q Exactive HF mass spectrometer (Thermo Fisher, Bremen, Germany). Intact masses were measured at resolution 70,000 (at *m*/*z* 200) in the orbitrap using an automatic gain control (AGC) target value of 3E6 charges. The top 15 peptide signals (charge-states 2^+^ and higher) were submitted to MS/MS in the higher-energy collision dissociation(HCD) cell (1.6 amu isolation width, 25% normalized collision energy). MS/MS spectra were acquired at resolution 17,500 (at *m*/*z* 200) in the orbitrap using an AGC target value of 1E6 charges, a maxIT of 32 ms and an underfill ratio of 0.1%. Dynamic exclusion was applied with a repeat count of 1 and an exclusion time of 30 s.

MS/MS spectra were searched against the Swissprot FASTA file (release January 2018, 42,258 entries, canonical and isoforms) using MaxQuant 1.6.0.16. Enzyme specificity was set to trypsin and up to two missed cleavages were allowed. Cysteine carboxamidomethylation was treated as fixed modification and methionine oxidation and protein N-terminal acetylation as variable modifications. Peptide precursor ions were searched with a maximum mass deviation of 4.5 ppm and fragment ions with a maximum mass deviation of 20 ppm. Peptide and protein identifications were filtered at an false discovery rate (FDR) of 1% using the decoy database strategy. The minimal peptide length was 7 amino acids. Proteins that could not be differentiated based on MS/MS spectra alone were grouped to protein groups (default MaxQuant settings). Searches were performed with the label-free quantification option selected. The mass spectrometry proteomics data have been deposited to the ProteomeXchange Consortium via the PRoteomics IDEntifications (PRIDE) partner repository (www.ebi.ac.uk/pride/archive), with the dataset identifier PXD015369 [26]. Proteins should be detected in at least 2 out of 3 replicates in one group. P values < 0.05 and fold change > 3 or < −3 were considered statistically significant and biologically relevant. Unsupervised clustering was performed using 1-Spearman correlation with complete linkage and supervised clustering was performed using Euclidean distance with complete linkage using R studio.

### 2.8. Functional Data Mining to Obtain Insight into Potential Resistance Mechanisms

#### 2.8.1. Identification of mRNA Targets

To select previously validated mRNA targets the bioinformatics algorithms miRTarBase (http://mirtarbase.mbc.nctu.edu.tw/php/index.php), DIANA-tools (algorithm TarBase v8 (http://diana.imis.athena-innovation.gr/DianaTools/index.php)) and miRDB [27] were used.

Selection criteria for miRTarBase were: 1) the target should be supported by strong experimental evidence i.e., western blot or reporter assay; 2) should be targeted by both miRNAs. Selection criteria for DIANA LAB targets were: 1) the mRNA should be targeted by both miRNAs; 2) evidence in at least two publications; and 3) prediction score of 0.800 or higher. Selection criteria for miRDB targets were: 1) the mRNA should be targeted by both miRNAs; 2) the target should have a Target Score above 85. These cut-offs were chosen to decrease the number of candidates.

#### 2.8.2. Gene Ontology, Networks, and Protein Function

Function and possible networks of the proteins were found using Uniprot (https://www.uniprot.org/) and STRING database (https://string-db.org/cgi/input.pl). Lists of proteins selected for STRING database analysis consisted of significantly downregulated or up-regulated proteins in cells that were more sensitive after transfection per miRNA mimic. For each individual cell line, the differentially expressed proteins were first corrected for proteins that were significantly up- or downregulated in the corresponding cell line transfected with the negative control synthetic cel-miR-39-3p. The remaining differentially expressed proteins were corrected for proteins that were significantly up- or downregulated in the microsatellite instable (MSI)/P53mutant DLD1 cell line in which increased sensitivity to chemotherapeutics was not observed after transfection. A detailed workflow on datamining for proteomics is presented in Figure S1.

#### 2.8.3. mRNA Target Site Analysis of Detected Proteins

Bioinformatics algorithms miRTarBase, DIANA-tools and miRDB were used to uncover supportive evidence for the most promising differentially expressed proteins after transfection with either miRNA. Each database was investigated for evidence of the most promising targets. If a target was mentioned in a database it was scored as “evidence”, regardless of the strength of this evidence. In addition, the 3′UTR regions of these proteins were downloaded using R-package biomaRt and investigated for matching seed sequences of miR-195-5p and miR-497-5p using R-package microRNA. Downloaded 3′UTR regions were verified with the Basic Local Alignment Search Tool, BLAST^®^ (https://blast.ncbi.nlm.nih.gov/Blast.cgi).

## 3. Results

### 3.1. Transfection with miRNA Mimics Results in Elevated Levels of miRNA Expression

**Figure 1:**
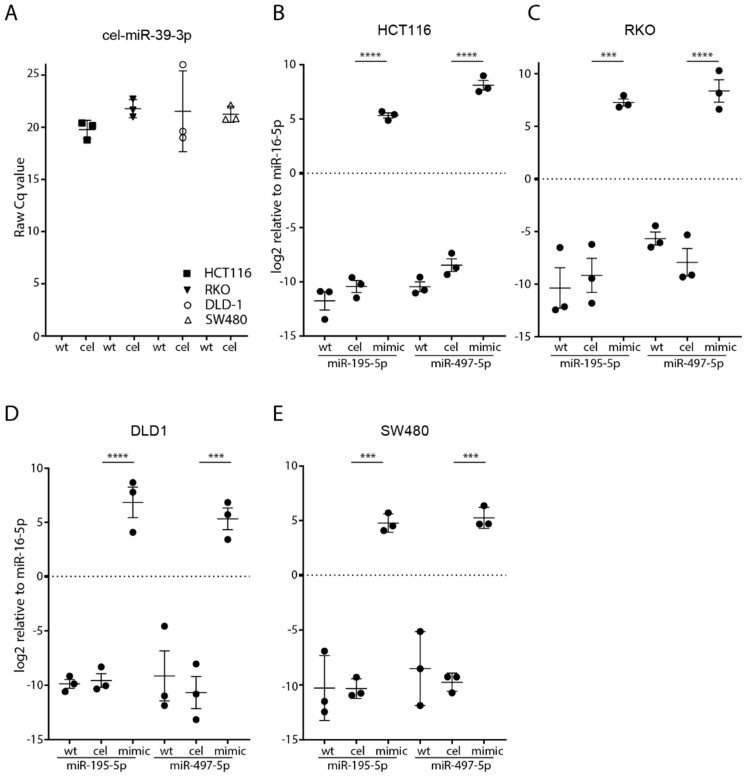
miRNA expression and transfection efficiency in CRC cells. Successful transfection of cel-miR-39-3p in the negative control transfected cells for all four different CRC cell lines (**A**). *y*-axis represents raw Cq value of cel-miR-39-3p. Successful transfection of miR-195-5p and miR-497-5p in the four different cell lines HCT116 (**B**), RKO (**C**), DLD1 (**D**) and SW480 (**E**). *y*-axis represents log2 expression of miR-195-5p and miR-497-5p relative to miR-16-5p (control). Data is presented as the mean of three independent transfection experiments (measured in duplicate) ± the standard error of the mean. *** *p* < 0.001, **** *p* < 0.0001, wt: wild-type, cel: cel-miR-39-3p negative control transfection, mimic: expression level 48 h after transfection.

No expression of cel-miR-39-3p was observed in wild-type control cell lines; however, it was detected in all four cel-miR-39-3p transfected cells (range 18.6–25.6 raw Cq value) (Figure 1A). Quantification of miRNA expression showed low expression levels of miR-195-5p and miR-497-5p in all four human CRC cell lines HCT116, RKO, DLD-1 and SW480 prior to transfection (Figure 1B–E). Significantly increased expression of miRNAs was observed after transfection with miRNA mimics in all CRC cell lines, while no difference was observed between the wild-type cell lines and after transfection with a negative control (cel-miR-39-3p) (Figure 1B–E). After transfection with miR-195-5p mimic, its increased expression ranged between 15.1 and 16.4 log_2_ Cq value (*p* < 0.01) for all four cell lines.

Increased expression of miR-497-5p ranged between 15.0 and 16.6 (*p* < 0.01) log_2_ Cq value for all four cell lines (Figure 1B–E). These findings indicate that transfection with miRNA mimics successfully generated an increased miRNA expression in all of the CRC cell lines.

### 3.2. Increasing Sensitivity to Chemotherapy after Transfection with miRNA Mimics

#### 3.2.1. Increased Sensitivity to Oxaliplatin in MSI/P53wt HCT116 Cells

**Figure 2:**
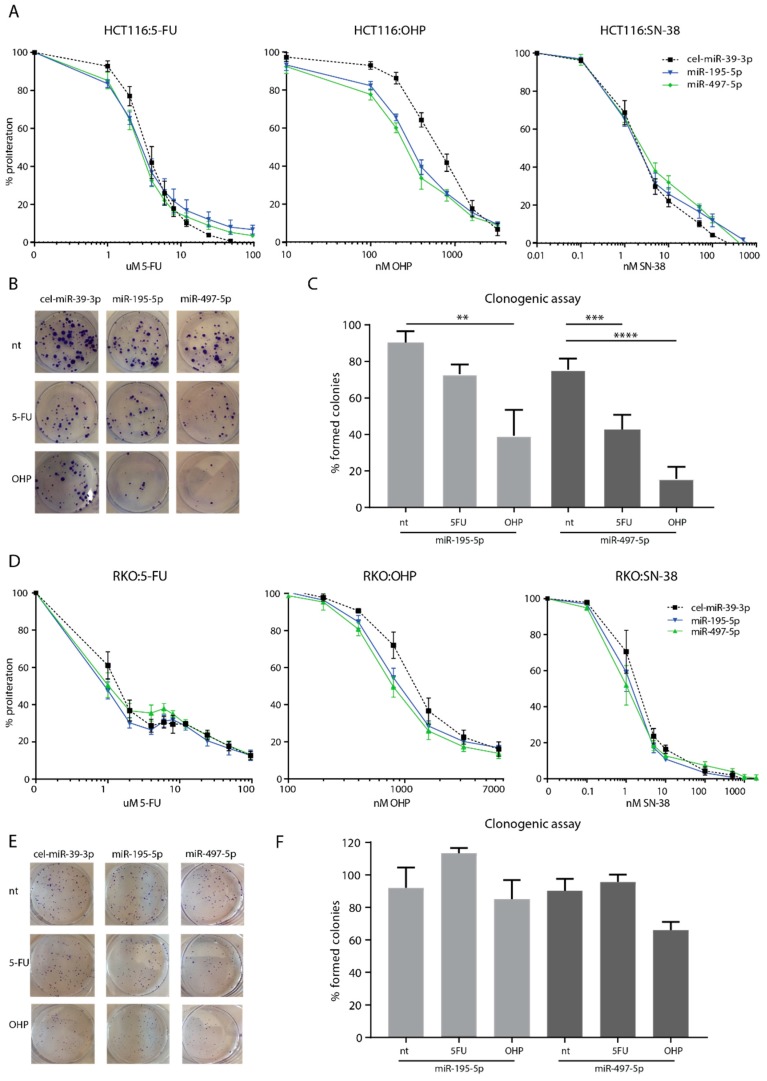
HCT116 and RKO cells after transfection with miR-195-5p or miR-497-5p mimic. MTT sensitivity assays for HCT116 cells (**A**) and RKO cells (**D**) were performed in triplicate in three independent experiments and are presented as average % proliferation compared to the proliferation of a non-treated control triplicate ± the standard error of the mean (SEM). Presentation of a single clonogenic assay experiment of HCT116 (**B**) and RKO (**E**). Bar graphs of the percentage of formed colonies compared to the negative control transfection (cel-miR-39-3p) of HCT116 cells (**C**) and RKO cells (**F**) presented as averages of duplicate colony counts from three independent experiments ± SEM. 5-FU; 5-fluorouracil, OHP; oxaliplatin, SN-38; irinotecan, nt; no treatment, ** *p* < 0.005, *** *p* < 0.001, **** *p* < 0.0001.

HCT116 cells transfected with miR-195-5p and miR-497-5p showed a minor increase in sensitivity to treatment with 5-FU. The IC_50_ decreased from 5200 nM (negative control) to 3033 nM (miR-195-5p) and 3367 nM (miR-497-5p) (*n* = 3, Figure 2A). However, these differences were not significant (*p* = 0.16 and *p* = 0.24, respectively). Transfected HCT116 cells showed a significant increase in sensitivity to oxaliplatin: a decrease in the IC_50_ from 787 nM (negative control) to 313 nM (miR-195-5p, *p* < 0.01) and to 267 nM (miR-497-5p, *p* < 0.01) was observed (*n* = 3, Figure 2A). No increased sensitivity in HCT116 transfected cells was observed for irinotecan, IC_50_ 1.9 nM (*n* = 3, Figure 2A). Clonogenic assays revealed that HCT116 transfected cells formed 17% (miR-195-5p, *p* = 0.27) and 33% (miR-497-5p, *p* < 0.01) fewer colonies when treated with 5-FU (*n* = 3, Figure 2B,C). When treated with oxaliplatin, HCT116 formed significantly fewer colonies after transfection with miR-195-5p (51% (*p* < 0.01)) and miR-497-5p (60% (*p* < 0.01)) mimics, (*n* = 3, Figure 2B,C). Clonogenic assays showed no formation of colonies after treatment with the IC_50_ of irinotecan (1.9 nM) under all conditions and are thus inconclusive. For this reason, no further clonogenic assays using irinotecan were performed.

#### 3.2.2. Mild Increased Sensitivity to Oxaliplatin of MSI/P53wt RKO Cells

No differences in sensitivity to 5-FU or irinotecan were found after transfection with either mimic, with a consistent IC_50_ of 1000 nM for 5-FU and 1 nM for irinotecan (Figure 2D). RKO transfected cells showed an increased sensitivity to oxaliplatin with a decreased IC_50_ from 1223 nM (negative control) to 873 nM (miR-195-5p, *p* = 0.10) and to 823 nM (miR-497-5p, *p* = 0.06) (Figure 2D). Clonogenic assays revealed that RKO cells formed a similar number of colonies after transfection with miR-195-5p mimic, and 20% fewer colonies (*p* = 0.20) when treated with oxaliplatin after transfection with miR-497-5p mimic (Figure 2E,F).

#### 3.2.3. No Increased Sensitivity to Chemotherapy in MSI/P53mut DLD-1 Cells and Microsatellite Stable (MSS)/P53wt SW480 Cells

MTT cell viability and clonogenic assays showed no effect on the sensitivity to 5-FU, oxaliplatin or irinotecan in DLD-1 and SW480 cells after transfection with either mimic (Figure S2A–E).

### 3.3. Target Inhibition after miR-195-5p or miR-497-5p Mimic Transfection in MSI Cell Lines HCT116 and DLD-1

**Figure 3:**
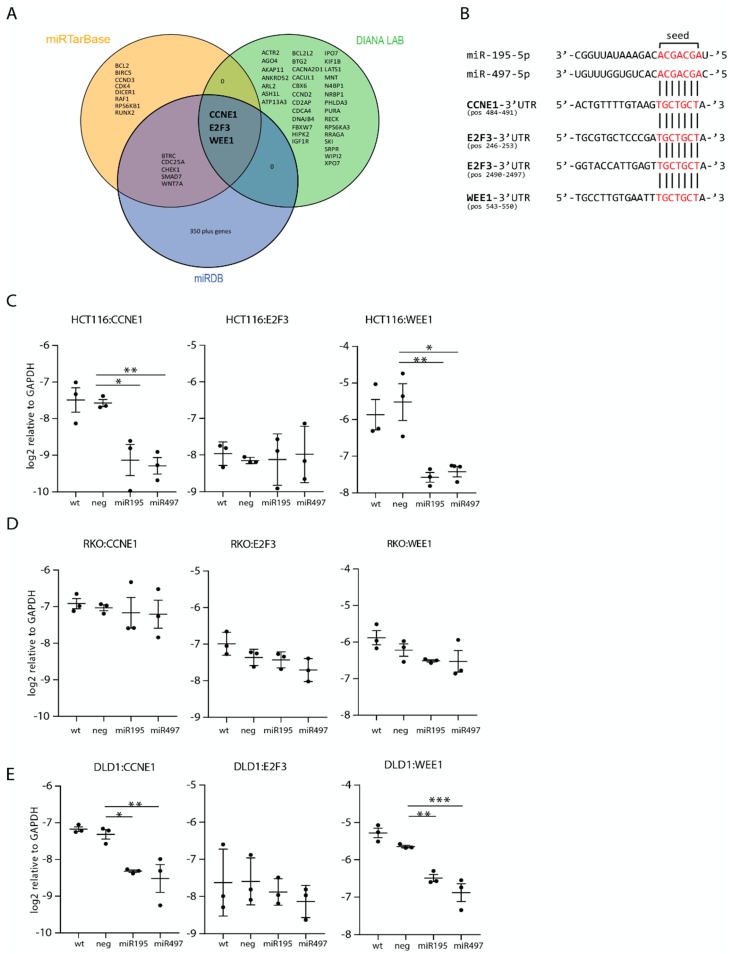
miR-195-5p and miR-497-5p target selection and quantification. (**A**) Venn diagram of the targets selected with miRTarBase, DIANA LAB, and miRDB with the coinciding target mRNAs of the three databases presented in the center. (**B**) Mature miRNA sequence of miR-195-5p and miR-497-5p matched to the 3′UTR target region of the selected targets with the seed sequence of each miRNA shown in red. Expression levels, presented as log_2_ relative to GAPDH of the specific mRNA targets CCNE1, E2F3, and WEE1, measured with RT-qPCR in HCT116 cells (**C**), RKO cells (**D**) and DLD-1 cells (**E**). Each target for each transfection is quantified in duplicate in three independent experiments, presented as average ± the SEM. wt: wild-type non-transfected control, neg: negative control transfection (cel-miR-39-3p). * *p* < 0.05, ** *p* < 0.01, *** *p* < 0.001.

Three targets; CCNE1, WEE1 and E2F3 were identified by all algorithms (Figure 3A). Mature sequences of miR-195-5p, miR-497-5p and mRNA 3′-UTR regions of these targets demonstrate a highly conserved binding site of the seed sequence for both miRNAs (Figure 3B). RT-qPCR quantification showed that CCNE1 and WEE1 were significantly lower expressed in HCT116 miR-195-5p mimic transfected cells with a decrease in log2 Cq value of 1.6 (*p* = 0.01) and 2.1 (*p* < 0.01), respectively (Figure 3C). For HCT116 miR-497-5p mimic transfected cells, a log2 decreased expression of 1.7 (*p* < 0.01) was found for CCNE1 and 1.9 (*p* = 0.01) for WEE1. mRNA expression levels in DLD-1 transfected cells decreased with 1.0 (CCNE1, *p* = 0.02) and 0.8 (WEE1, *p* < 0.01) Cq value for miR-195-5p. In miR-497-5p mimic transfected DLD-1 cells mRNA levels decreased by 1.2 Cq value for both CCNE1 (*p* < 0.01) and WEE1 (*p* < 0.01). E2F3 showed no differential expression in all three CRC cell lines. None of the selected targets were downregulated after transfection with either mimic in RKO and SW480 cells (Figure 3D and Figure S2F). These results indicate that miR-195-5p and miR-497-5p miRNA mimics are further processed into functional molecules after transfection into CRC cells.

### 3.4. Proteomic Analysis

#### 3.4.1. Proteomic Analysis for Detection of Potential Targets Involved in Chemotherapy Resistance in CRC Cells

In total, 4623 proteins were detected at least once in the four different cell lines. Unsupervised cluster analysis of the detected proteins revealed two clusters (HCT116/RKO combined and SW480/DLD-1 combined). All four cell lines clustered together, indicating reproducible results for each condition. In the RKO and HCT116 cell lines, miR-195-5p, and miR-497-5p mimic transfected cells clustered separately from the wild-type and negative control transfected cells. As for the SW480 and DLD-1 cells, transfected and non-transfected cells were mixed (Figure S3).

#### 3.4.2. Differential Proteins after Transfection with Negative Control cel-miR-39-3p

A minor effect on the global proteome of all four CRC cell lines was observed after transfection with cel-miR-39-3p (Figure S4). A paired comparison of wild-type versus cel-miR-39-3p transfections identified the most differentially expressed proteins in RKO cells (*n* = 45, 1.0%) and the least in SW480 cells, (*n* = 28, 0.6%) (*n* = 3, *p* < 0.05, fold change > 3 or < −3 (Table 1)).

#### 3.4.3. Differential Proteins after Transfection with miR-195-5p or miR-497-5p Mimics

**Figure 4:**
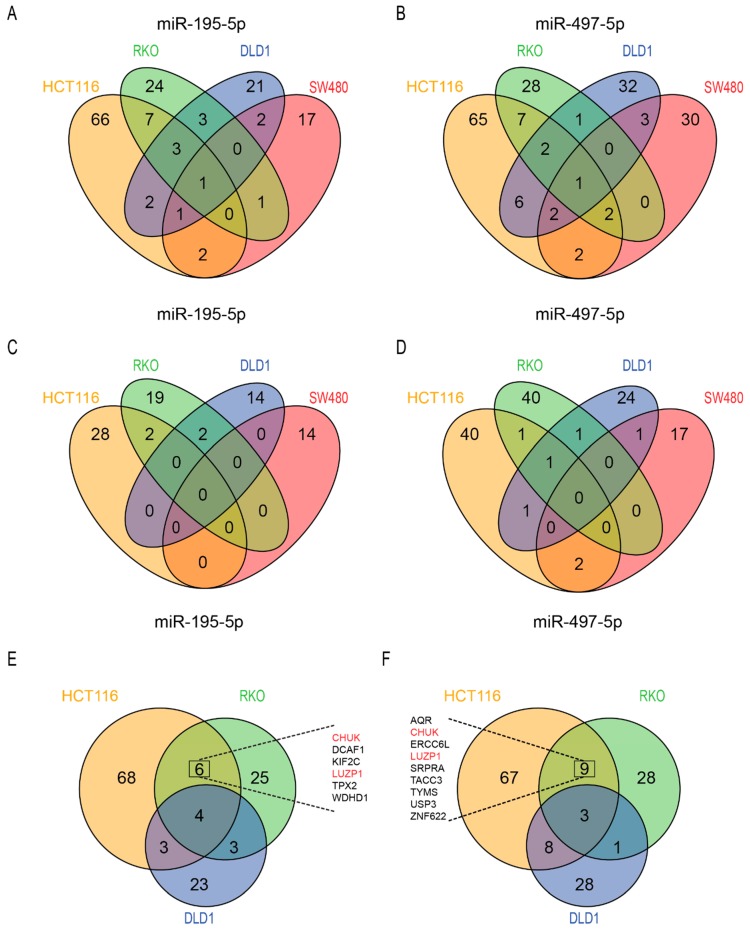
Number of significantly differential expressed proteins after transfection with miR-195-5p and miR-497-5p miRNA mimics. Venn diagram of the downregulated proteins in the four cell lines after transfection with mimics of miR-195-5p (**A**) and miR-497-5p (**B**). Venn diagram of the upregulated proteins in the four cell lines after transfection with mimics of miR-195-5p (**C**) and miR-497-5p (**D**). Venn diagram of overlapping downregulated proteins in MSI CRC cells after transfection with mimics of miR-195-5p (**E**) and miR-497-5p (**F**). Coinciding significantly differential expressed proteins in MSI/P53 wt CRC cells transfected with miR-195-5p mimic and miR-497-5p mimic are listed in (**E**) and (**F**) respectively. In red two proteins downregulated in all four conditions.

Supervised clustering on differential expressed proteins in miRNA-mimic transfected cells showed that miR-195-5p mimic transfected cells clustered together with miR-497-5p mimic transfected cells (Figure S5). After correction for the negative control transfection, most up- and downregulated proteins were identified in HCT116 cells transfected with miR-497-5p (Table 1). For both miRNAs most overlapping downregulated proteins were identified in HCT116 and RKO cells (Figure 4A,B). Two overlapping proteins were upregulated in HCT116 and RKO and in HCT116 and SW480 after transfection with miR-195-5p mimic, and two proteins were upregulated in HCT116 and SW480 after transfection with miR-497-5p mimic (Figure 4C,D). To identify proteins related to chemotherapy sensitivity, overlapping downregulated proteins in the MSI/P53wt cell lines that showed increased sensitivity to 5-FU and/or oxaliplatin after transfection with either miRNA mimic (HCT116 and RKO) were analyzed. Six overlapping downregulated proteins were found after transfection with miR-195-5p mimic (Figure 4E). Nine overlapping proteins were found after transfection with miR-497-5p mimic (Figure 4F). Two proteins, CHUK and LUZP1, were downregulated in HCT116 and RKO cells after transfection of both miRNA mimics (Figure 4F). All up- and downregulated proteins are presented in Table S2.

### 3.5. Target Analysis of Overlapping Downregulated Proteins in RKO and HCT116 Cells

**Figure 5:**
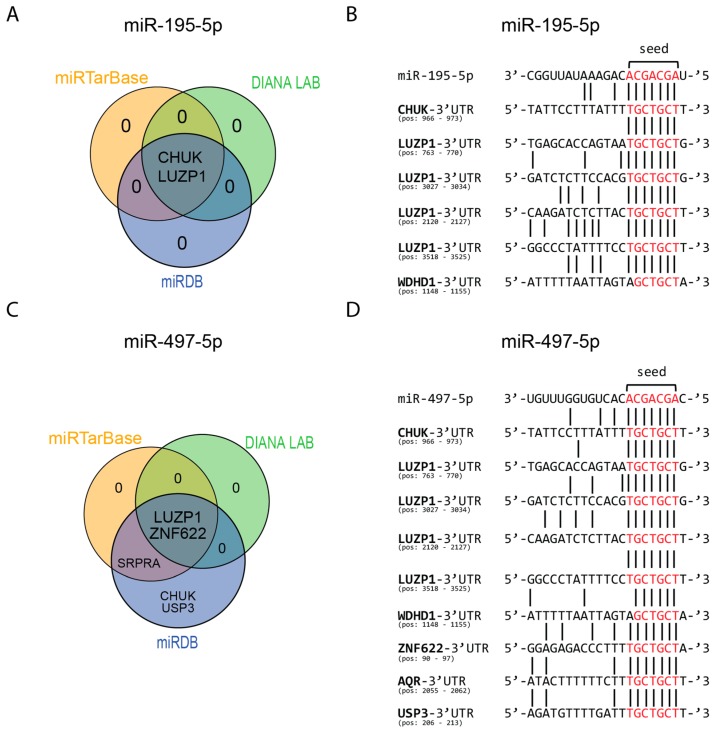
miRNA target evidence of differential expressed proteins from the proteomics screen. Venn diagram of mRNA target evidence found in the different databases for miR-195-5p (**A**) and miR-497-5p (**C**). mRNA target sites of potential targets of miR-195-5p (**B**) and miR-497-5p (**D**) based on the seed sequence (given in red).

Transfection of RKO and HCT116 cells with miR-195-5p and miR-497-5p mimics revealed six and nine overlapping downregulated proteins respectively (Figure 4E,F). Analysis in the three databases for mRNA targets of miR-195-5p revealed evidence for two (CHUK and LUZP1) overlapping downregulated proteins. The other four proteins were not found in any of the three databases (Figure 5A). 3′UTR target site investigation revealed matching mRNA target sites for three out of six proteins (CHUK, LUZP1, and WDHD1). For LUZP1 four matching targets at different positions were found in its 3′UTR region (Figure 5B).

Evidence for six overlapping downregulated proteins by miR-497-5p mimic was found in at least one database. LUZP1 and ZNF622 were found in all three databases (Figure 5C). As miR-195-5p and miR-497-5p have similar seed sequences, similar mRNA target sites were found for CHUK, LUZP1, and WDHD1. One mRNA target site was found for ZNF622, RNA helicase aquarius (AQR) and USP3 (Figure 5D). One protein, SRPRA, had target site evidence in two databases; however, no potential mRNA target site for this protein was found in its 3′UTR region.

### 3.6. Protein Function Analysis of Oxaliplatin Sensitivity in MSI/P53wt CRC Cell Lines

Protein-protein interaction (PPI) analysis was performed on differentially expressed proteins of the sensitized cell lines using the STRING database. Overlapping differentially expressed proteins between HCT116 or RKO (MSI, P53wt) and DLD-1 cells (MSI, P53mut) were excluded from this analysis.

Analysis of the 107 downregulated proteins after transfection with miR-195-5p mimic revealed a significant functional relation of 6 proteins in the cell-cycle KEGG pathway (FDR = 0.01), namely: BUB1B, CCNB1, CDC20, CDK4, CREBBP, and PLK1. STRING database analysis of the 116 downregulated proteins after miR-497-5p mimic transfection in HCT116 and RKO cells revealed a significant functional relation of six proteins in the pyrimidine metabolism KEGG pathway (FDR < 0.05), namely: NT5C3A, POLR1A, PRIM1, PRIM2, and TYMS.

STRING database analysis revealed three significant KEGG pathways related to the upregulated proteins after transfection with miR-195-5p mimic, namely: B cell receptor signaling pathway (FDR = 0.04), T cell receptor signaling pathway (FDR = 0.01) and Alzheimer’s disease (FDR = 0.04). After transfection with miR-497-5p mimic, no KEGG pathways were significantly related to upregulated proteins.

Biological process analysis in STRING database revealed 160 processes that were significantly related to the downregulated proteins after transfection with either miRNA mimic (FDR < 0.05). The top 25 of these processes are listed in Table 2. The most significant biological processes related to these differentially expressed proteins were cell-cycle and cell-cycle division. Among these top 25, nine processes were involved in mitosis during the mitotic cell-cycle (Table 2). Fifty-nine biological processes were significantly related to the upregulated proteins (FDR < 0.05), of which the most significant were related to cellular component organization (data not shown). Overall, this PPI analysis indicates the involvement of cell-cycle proteins in the sensitivity of MSI/P53wt CRC cells to oxaliplatin.

## 4. Discussion

While the majority of patients with mCRC are treated with chemotherapy, acquired and intrinsic drug resistance causes major limitations in the outcome for patients. To optimize treatment, new strategies were investigated to overcome this hurdle using a miRNA mimic-based approach, as miRNAs are emerging as new therapeutics. Initially a model was established in which increased miR-195-5p and miR-497-5p expression sensitized CRC cancer cells to chemotherapy. In addition, global proteomic analysis was performed to reveal possible underlying mechanisms to increase sensitivity to oxaliplatin chemotherapy. This approach revealed a list of specific proteins involved in cell division, DNA damage response and nuclear factor kappa-B signaling, which are promising targets to potentiate chemotherapy in treatment naïve MSI/P53wt CRC cells. In addition, protein LUZP1, to our knowledge not related to the pathways mentioned above, was downregulated by miR-195-5p and miR-497-5p mimic transfection in both MSI/P53wt CRC cell lines. LUZP1 downregulation was associated with increased sensitivity to oxaliplatin.

In this study, an increase in sensitivity to oxaliplatin and a mild increase in sensitivity to 5-FU was observed after elevated expression of both miR-195-5p and miR-497-5p in treatment naïve CRC cells, which is in line with results of previous studies [15,28]. For miRNA replacement therapy as strategy to sensitize cells to chemotherapy a model with treatment naïve non-resistant cell lines was used, enabling the possibility to study intrinsic chemotherapy resistance mechanisms. After transfection with miR-195-5p and miR-497-5p mimics increased sensitivity was only observed in HCT116 and RKO cells and not in DLD-1 and SW480 cells. Interestingly, both cell lines that showed increased sensitivity were MSI and P53wt. No effect was observed in the MSI/P53mut DLD-1 cells and MSS/P53mut SW480 cells. Feng et al. previously described a sensitizing effect of increased miR-195-5p expression in SW480 cells resistant to 5-FU [28]. This may suggest that the P53 mutation status combined with the MSI phenotype in CRC is involved in intrinsic resistance mechanisms to 5FU. Though, a limitation to support this hypothesis is the lack of results of a negative control consisting of an MSS/P53wt CRC cell line.

### 4.1. WEE1 and CCNE1

To further examine biological activity of transfected miRNAs, mRNA targets were predicted from three independent miRNA databases using specific selection criteria and confirmed WEE1 and CCNE1 inhibition after miRNA-mimic transfection. Both WEE1 and CCNE1 are involved in cell-cycle progression. Previous studies have shown that WEE1 inhibition is associated with increased sensitivity to platinum-based therapies and possibly related to intrinsic resistance [17]. As a response to DNA damage WEE1 inhibition stimulates G_2_ to M phase transition without DNA repair resulting in cell death. P53 deficient cancer cells often lack a functional G_1_ checkpoint, and rely on G_2_ checkpoint for DNA repair. Therefore, a combination of WEE1 inhibition and chemotherapy may have a synergistic effect in P53 deficient cancer cells. In this study, WEE1 was downregulated after transfection with miRNA mimics in MSI HCT116 and DLD-1 cells. As DLD-1 cells are P53mut, it was expected that these cells would become more sensitive to chemotherapy after miRNA replacement. On the contrary, HCT116 and RKO, both P53wt, showed increased sensitivity to chemotherapy. This is in line with studies describing a sensitizing effect of WEE1 independent of P53 functionality [29]. This suggests that other mutations, besides P53, involved in the G_1_ to M checkpoint are responsible for the sensitizing effect of WEE1. For HCT116 this may be aberration of CDKN2A as suggested by Geenen and colleagues (Table S1) [30].

### 4.2. CHUK

One overlapping protein, CHUK, identified as differentially expressed in chemotherapy sensitized cells could be related to nuclear factor kappa-B (NF-κB) signaling. CHUK, also known as IKK-α, is part of the IKK complex and may be activated by stimuli including DNA damage. Activated CHUK phosphorylates inhibitors of NF-κB leading to degradation of these inhibitors and subsequently translocation of free NF-κB into the nucleus. As a result, gene transcription of many different biological processes is activated including protection against apoptosis. DNA damage induced by oxaliplatin may activate CHUK and in turn rescue damaged cells from apoptosis by NF-κB signaling. Therefore, negative regulation of NF-κB through inhibition of CHUK by miR-195-5p or miR-497-5p possibly could sensitize colorectal cancer cells to chemotherapeutic agents. Interestingly, UBE2D2, an inhibitor of NF-κB alpha was found among the significantly upregulated proteins in the proteomics analysis after transfection with miR-195-5p mimic. This may be a direct effect of reduced CHUK levels. Inhibition of CHUK by miR-195-5p replacement has also been described by Ding et al. [31]. Furthermore, Zhao and colleagues recently found evidence for CHUK as a target of miR-15b-5p [32]. miR-15b-5p belongs to the miR-15 precursor family which also includes miR-195-5p and miR-497-5p. This family has a similar conserved seed sequence and consequently similar potential targets. Comparable to our results, increased expression of miR-15b-5p resulted in reduced CHUK levels and sensitized CRC cells to 5-FU. Moreover, Jani et al. described a synergistic effect of a selective CHUK inhibitor combined with oxaliplatin in CRC cells [33].

### 4.3. ZNF622 and USP3

Many inhibited proteins in the cells that were sensitized after miRNA-mimic transfection could be related to cell-cycle/cell division. Of these, two overlapping proteins, ZNF622 and USP3, were found differentially expressed after transfection with miR-497-5p mimic. Zinc finger protein 622 (ZNF622) is a coactivator of MYBL2 which is a protein required for cells to progress into S-phase [34]. Lin et al. presented evidence that high expression levels of MYBL2 can bypass P53-induced G_1_ cell-cycle arrest [35]. As an intact P53 pathway is present in HCT116 and RKO cells, reduced expression levels of MYBL2 through ZNF622 inhibition by miR-195-5p or miR-497-5p may result in an increased P53-induced G_1_ cell-cycle arrest. Moreover, inhibition of G_1_ to S-phase transition has previously been associated with enhanced oxaliplatin cytotoxicity [36].

Ubiquitin carboxyl-terminal hydrolase 3 (USP3), is a member of the ubiquitin-proteasome system regulating many cellular processes, including cell-cycle progression. Fang et al. found that knockdown of USP3 resulted in reduced levels of cyclin D and E leading to reduced number of colonies in gastric cancer cells suggesting involvement of USP3 in G_1_ to S-phase progression. Also, overexpression of USP3 has been associated with gastric cancer tumorigenesis [37]. Inhibition of USP3 by increased miR-195-5p or miR-497-5p expression may result in G_1_ arrest of the cells and subsequently enhanced sensitivity to chemotherapy.

In addition, proteomic analysis revealed that 9 out of the top 25 identified biological processes were associated with mitosis. As such, the majority of the proteins that were downregulated by increased miR195-5p and miR-497-5p expression could be related to regulation of mitosis, including key regulators of mitosis PLK1 (Table S2) and TPX2 (Figure 4) [38]. Essential regulator of G_1_ to S-phase transition, CDK4, and regulator of G_2_ to M transition, cyclin-B1, were downregulated in RKO or HCT116 cells, suggesting that these cells may be in a mixed G_1_ or G_2_ arrest after transfection with either miRNA mimic, and therefore potentiating sensitivity to oxaliplatin. G_1_ or G_2_ arrest, due to increased expression of miR-195-5p or miR-497-5p, may explain why proteins KIF2C, TPX2, TACC3, and ERCC6L, all involved in mitosis, are downregulated without additional target evidence. Interestingly, both KIF2C and TPX2 are overexpressed in CRC and have been associated with a poor prognosis [39,40].

TACC3 overexpression is related to chemoradiotherapy resistance in patients with locally advanced rectal cancer [41]. Inhibition of TACC3 in breast cancer induced apoptotic cell death [42]. Schneider et al. related TACC3 inhibition induced apoptosis to caspase-dependent cell death, in particular by activation of caspase-3 [43]. Accordingly, caspase-3 was detected among the upregulated proteins in the present study, suggesting increased apoptotic activity after restoring expression of miR-195-5p or miR-497-5p using mimics.

### 4.4. WDHD1 and AQR

WDHD1 and AQR were both overlapping downregulated proteins in sensitized cells after transfection with miR-195-5p or miR-497-5p mimics, respectively. Both proteins are possibly related to DNA damage response. WDHD1, WD repeat, and HMG-box DNA-binding protein 1 or And-1, is essential for the stability of histone acetyltransferase and generally controls non-depressible 5 (Gcn5). Gcn5 is known to acetylate multiple lysines including histone H3 lysate 9 and lysate 56. Depletion of WDHD1 in HCT116 cells resulted in reduced Gcn5 expression and subsequently reduced H3K9 and H3K56 acetylation [44]. Recent work has revealed that H3K9 and H3K56 are acetylated at sites of DNA damage and involved in activating cell-cycle checkpoints for efficient DNA repair including P53 [45,46]. P53 wild-type cells with impaired H3K9 and/or H3K56 acetylation might be more sensitive to genotoxic agents due to reduced ability to repair DNA damage [45,47]. Inhibition of WDHD1 possibly sensitizes CRC cells by reducing DNA damage tolerance, which could explain why HCT116 and RKO cells are more sensitive for chemotherapy after increased miR195-5p or miR-497-5p expression.

AQR is involved in homologous recombination-mediated repair of DNA double strands breaks. Knockdown of AQR sensitized cancer cells for DNA damaging agents such as mitomycin C, camptothecin and cisplatin probably by reducing DNA damage tolerance [48].

### 4.5. LUZP1

Leucine zipper protein 1 (LUZP1) has many different predicted 3′UTR target sites and is therefore most likely inhibited due to increased miR-195-5p or miR-497-5p expression. LUZP1 is predominantly expressed in the brain, where it is suggested to be involved in embryonic brain development. LUZP1 knock out resulted in elevated apoptosis of mesenchymal cells in mice [49]. In humans, high mRNA levels of LUZP1 have been observed in the colon (http://bioinfo.wilmer.jhu.edu/tiger/). More recently, Wang and colleagues addressed LUZP1 as a new actin-binding protein, particularly involved in actin cross-linking [50]. Actin cross-linking factors play a role in coordination of migration and proliferation. Knockdown of microtubule cross-linking factor 1 (MCAF1) inhibits the cell-cycle at S-phase and reduces cell proliferation [51]. Consequently, this may lead to G_1_ arrest and increased sensitivity to chemotherapy. In glioblastoma inhibition of MCAF1 resulted in reduced wnt signaling mediators Axin1 and β-catenin [51]. Hyperactivation of wnt is a common event in CRC and drugs targeting β-catenin mediated transcription in CRC are currently under investigation [52]. As MCAF1 for glioblastoma, LUZP1 may have a similar role in CRC and inhibition may reduce β-catenin signaling. However, evidence of LUZP1 functioning as actin cross-linking protein is largely lacking and more research is necessary to elucidate the importance of LUZP1 as a target to sensitize CRC cells to chemotherapy.

Despite the interesting results that transfection with miRNA mimics of miR-195-5p and miR-497-5p increased sensitivity to oxaliplatin in MSI/P53wt CRC cells, this study has several limitations. It should be noted that the identified targets in this study were found by proteomics or by RT-qPCR. Unfortunately, WEE1 and CCNE1 were not validated using the proteomics data set.

On the other hand, the downregulated proteins identified with proteomics were not validated on RNA expression level using RT-qPCR. Inhibition of the identified targets by these miRNA mimics should be further confirmed by more comprehensive functional analysis of each individual protein to unravel the role of the proteins in the resistance mechanism. Another limitation is the use of 2D cell cultures only. Recent advances in cell culture techniques allows for more complex 3D cell cultures, as for example organoids, which could be more representative of a tumor compared to a 2D model. The tumor microenvironment, also an important player in therapy resistance, could be included in such 3D models. Another limitation is the lack of experiments to confirm if the observed effect is truly MSI/P53wt specific. As the identified targets were not downregulated by miR-195-5p and miR-497-5p in the MSS and P53mut cells, it could be possible that these cells would become more sensitive to chemotherapy by specific inhibition of the identified protein targets by other biological mechanisms, which have not been further explored in the present study.

In summary, this study showed that restoring downregulated miRNA expression by miRNA mimics could increase sensitivity to oxaliplatin in MSI/P53wt CRC in vitro. Proteomics analysis on CRC cell lines after transfection with mimics of miR-195-5p and miR-497-5p revealed multiple biological processes that may be involved in sensitivity to chemotherapy. This emphasizes the possibility that a single miRNA inhibits multiple targets in a single cell, and may be effective as a combination therapy by itself. The data presented here provides evidence that miRNA-based therapeutics combined with chemotherapy may be a promising strategy to improve therapy efficacy for patients with mCRC.

## Figures and Tables

**Table 1:** Numbers of differentially up- or downregulated proteins after transfection of each miRNA mimic. For miR-195-5p and miR-497-5p numbers represent significant up- or downregulated proteins compared to the cells transfected with cel-miR-39-3p (*p* < 0.05, fold change > 3 or < −3). For cel-miR-39-3p numbers represent significant up- or downregulated proteins compared to the non-transfected wild-type cells.

	miR-195-5p		miR-497-5p		cel-miR-39-3p	
	Down	Up	Down	Up	Down	Up
**HCT116**	79 (1.7%)	30 (0.6%)	87 (1.9%)	45 (1.0%)	16 (0.3%)	24 (0.5%)
**RKO**	38 (0.8%)	23 (0.5%)	41 (0.9%)	43 (0.9%)	24 (0.5%)	20 (0.4%)
**DLD-1**	34 (0.7%)	16 (0.3%)	47 (1.0%)	28 (0.6%)	14 (0.3%)	20 (0.4%)
**SW480**	23 (0.5%)	14 (0.3%)	39 (0.8%)	20 (0.4%)	16 (0.3%)	12 (0.3%)

**Table 2:** Top 25 biological processes related to the downregulated proteins. The first column represents the biological process. The second column is the number of downregulated proteins through increased miR195/497 expression in RKO or HCT116 cells in the dataset related to that process. In bold the processes involved in mitosis. FDR: False Discovery Rate.

Gene Ontology Term	#Proteins	FDR
cell-cycle	46	7.06 × 10^−14^
cell division	30	7.06 × 10^−14^
mitotic cell-cycle	32	6.12 × 10^−13^
mitotic cell-cycle process	30	1.42 × 10^−12^
cell-cycle process	36	4.10 × 10^−12^
**mitotic nuclear division**	**16**	**8.82 × 10^−11^**
**nuclear division**	**19**	**1.65 × 10^−09^**
**sister chromatid segregation**	**14**	**3.43 × 10^−09^**
cellular component organization or biogenesis	86	5.85 × 10^−09^
organelle organization	61	1.99 × 10^−08^
chromosome organization	32	2.56 × 10^−08^
**mitotic sister chromatid segregation**	**12**	**4.31 × 10^−08^**
**mitotic spindle organization**	**10**	**2.72 × 10^−07^**
**microtubule cytoskeleton organization involved in mitosis**	**11**	**2.72 × 10^−07^**
cytoskeleton organization	29	5.12 × 10^−07^
**mitotic spindle assembly**	**8**	**9.57 × 10^−07^**
cellular component biogenesis	50	1.13 × 10^−06^
cellular component organization	78	1.29 × 10^−06^
cell-cycle phase transition	15	1.85 × 10^−06^
**spindle organization**	**11**	**2.32 × 10^−06^**
**spindle assembly**	**9**	**2.62 × 10^−06^**
regulation of cell-cycle	30	3.57 × 10^−06^
cellular process	151	5.64 × 10^−06^
mitotic cell-cycle phase transition	14	6.70 × 10^−06^
regulation of cell-cycle process	22	1.20 × 10^−05^

## Supplementary Materials

The following are available online at https://www.mdpi.com/2073-4409/8/9/1111/s1, Figure S1: Workflow and datamining of proteomics data, Figure S2: Cell viability and clonogenic assays of DLD1 and SW480 cells after treatment with chemotherapy, and mRNA expression in SW480 cells, Figure S3. Unsupervised cluster analysis of detected peptides in CRC cells. Figure S4: Supervised cluster analysis of differentially expressed peptides between wild-type cells and negative control transfected cells. Figure S5: Supervised cluster analysis of differentially expressed peptides between negative control transfected cells and cells transfected with miR-195-5p and miR-497-5p mimics, Table S1: Cell line characteristics of RKO, HCT116, SW480, and DLD1, Table S2: Significant up- and downregulated peptides after transfection of CRC cells with miR-195-5p mimic and miR-497-5p mimic.
