# Supplementary material for: Proteomic Analysis of miR-195 and miR-497 Replacement Reveals Potential Candidates that Increase Sensitivity to Oxaliplatin in MSI/P53wt Colorectal Cancer Cells
Source: Cells. 2019 Sep 19;8(9):1111. doi: 10.3390/cells8091111 (PMC6770888; doi:10.3390/cells8091111)
Supplement: Supplementary file 1 [file cells-08-01111-s001.pdf]

Supplementary Figures: Proteomic analysis of miR-195 and miR-497 replacement reveals potential candidates that increase sensitivity to oxaliplatin in MSI/P53wt colorectal cancer cells

Supplementary Figure S1: Workflow and datamining of proteomics data.

Supplementary Figure S2: Cell viability and clonogenic assays of DLD1 and SW480 cells after treatment with chemotherapy, and mRNA expression in SW480 cells.

Supplementary Figure S3. Unsupervised cluster analysis of detected peptides in CRC cells.

Supplementary Figure S4: Supervised cluster analysis of differentially expressed peptides between wild-type cells and negative control transfected cells.

Supplementary Figure S5: Supervised cluster analysis of differentially expressed peptides between negative control transfected cells and cells transfected with miR-195-5p and miR-497-5p mimics.

Supplementary Table S1: Cell line characteristics of RKO, HCT116, SW480 and DLD1.

Supplementary Table S2: Significant up- and downregulated peptides after transfection of CRC cells with miR-195-5p mimic and miR-497-5p mimic.

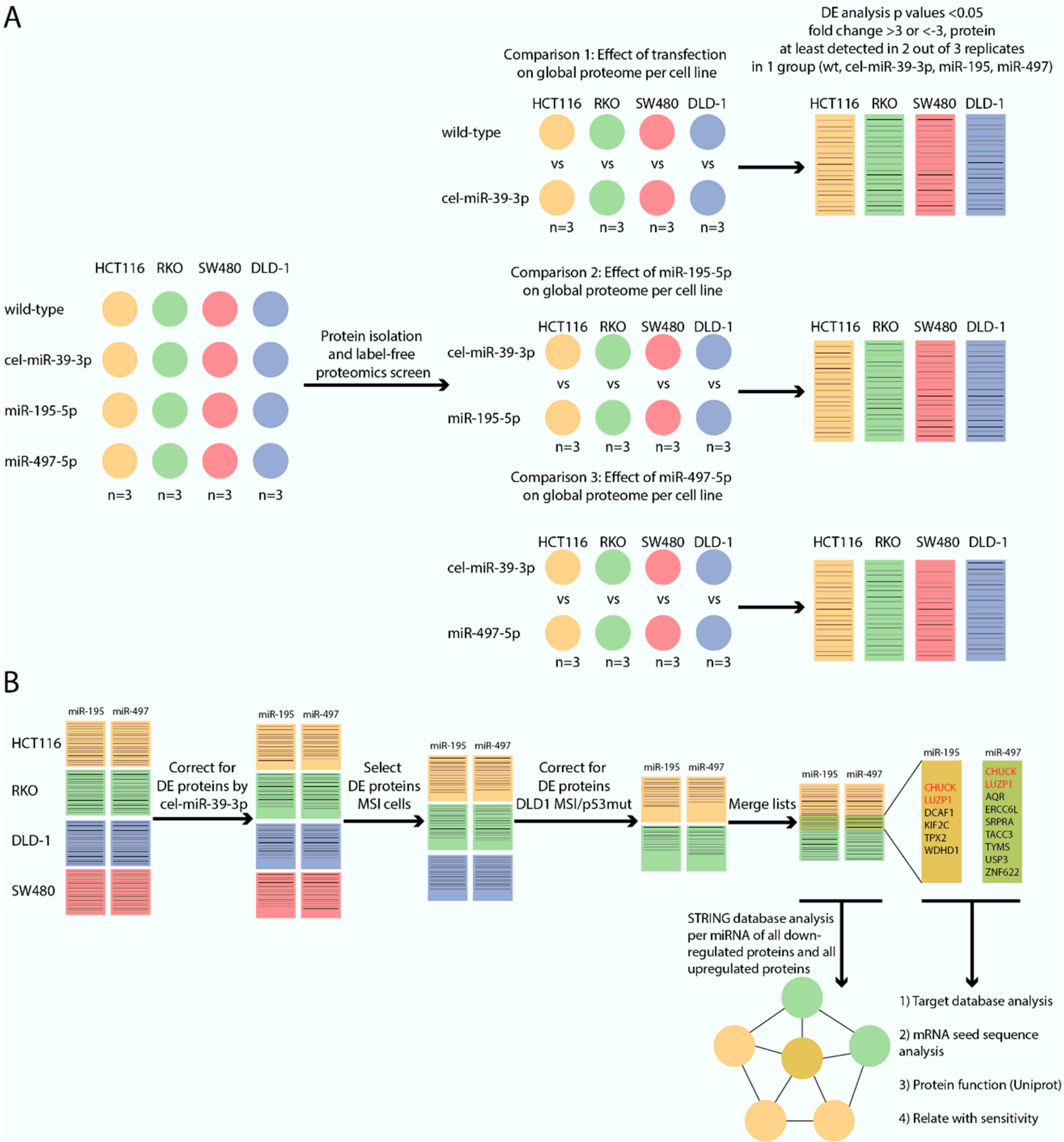

Supplementary Figure S1. Workflow and datamining of proteomics data. (**A**) Cell line transfections, number of replicates per cell line and comparisons for analyses of differentially expressed (DE) proteins. First, each cell line was transfected in triplicate with cel-miR-39-3p, miR-195-5p mimic and miR-497-5p mimic, in addition, triplicate non-transfected cells (wild-type) were included. Second, 48 hours after transfection proteins were isolated and a label-free proteomics screen was performed. Third, 3 comparisons were performed for all 4 cell lines to obtain DE proteins. Comparison 1: to identify the proteins DE by the transfection itself, comparison 2 and 3: to identify the proteins DE by transfection of miR-195-5p and miR-497-5p mimics respectively. (**B**) Corrections and comparison to retrieve relevant DE proteins. DE proteins were corrected for the DE proteins in comparison 1 for each cell line. Next, DE proteins in MSI cell lines were selected for further analysis. Thereafter, DE proteins in sensitized cells, RKO and HCT116, were corrected for DE proteins in MSI cell line DLD-1 (no increased sensitivity to chemotherapy). Next, DE proteins in HCT116 and RKO cells were merged per miRNA. Finally, two analyses were performed: 1) all DE proteins per miRNA were subjected for STRING database analysis to identify the most relevant involved biological processes and 2) overlapping DE proteins per miRNA were associated to drug sensitivity using multiple different approaches (1-4 in figure) DE; differentially expressed.

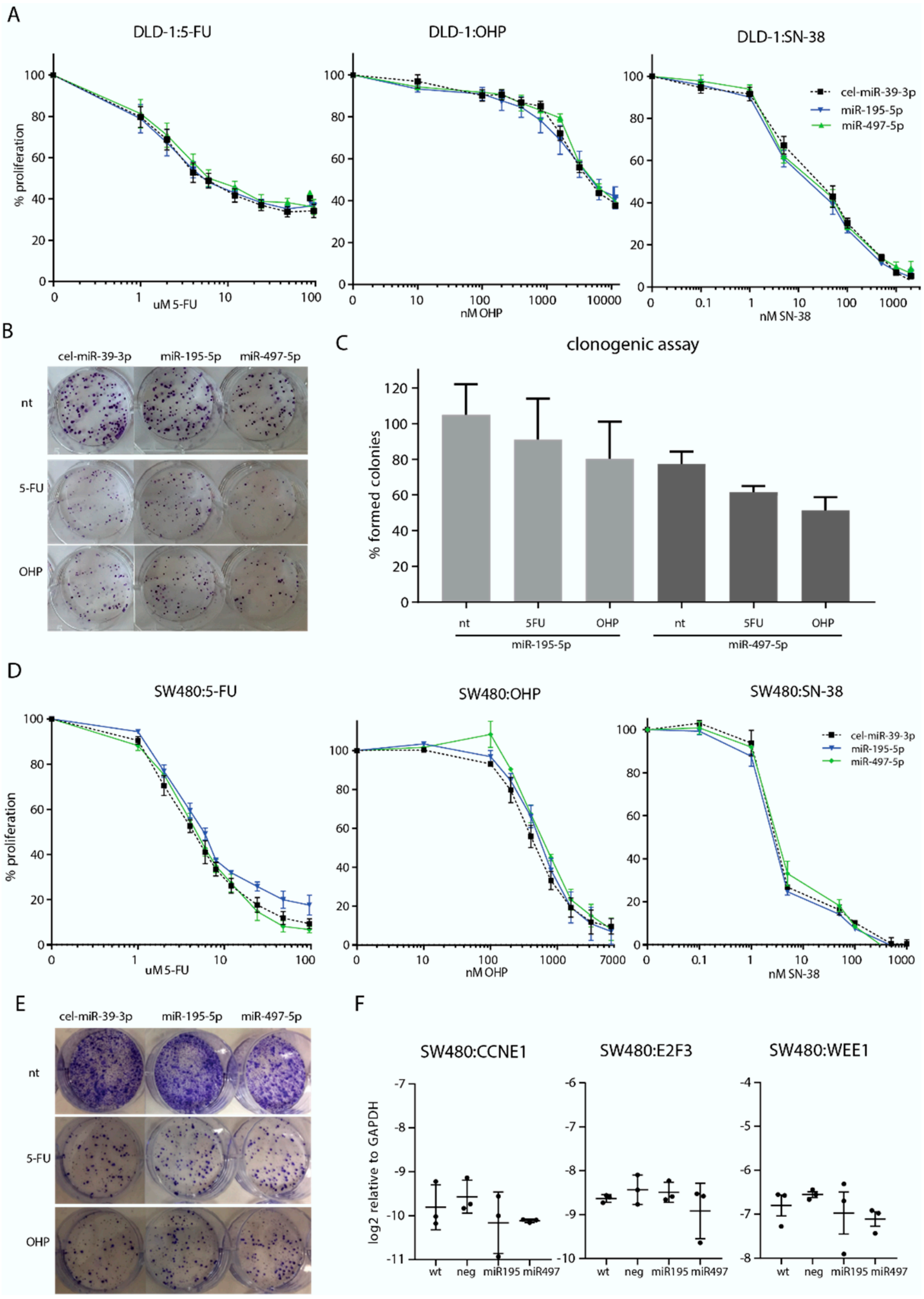

Supplementary Figure S2. Cell viability and clonogenic assays of DLD1 and SW480 cells after treatment with chemotherapy, and mRNA expression in SW480 cells. Cell viability assays were performed in three (DLD-1) and two (SW480) independent experiments in triplicate and are presented as average % proliferation compared to the proliferation of a non-treated control (± the standard error of the mean (SEM)): DLD-1 (**A**)**,** SW480 (**D**). Pictures of a single clonogenic assay experiment of DLD-1 cells (**B**) and SW480 cells (**E**). (**C**) Percentage of colonies after transfection with miR-195-5p and miR-497-5p mimics compared to the negative control transfection (cel-miR-39-3p). Bars represent the average of duplicate colony counts from three independent experiments of DLD-1 plus the SEM. As there were too many formed colonies in the non-treated SW480 cells these were not quantified. (**F**) Expression levels of selected mRNA targets relative to the expression of GAPDH in SW480 of wild-type and transfected cells**.** Each target is quantified in duplicate in three independent experiments, presented as average ± the standard error of the mean (SEM). 5-FU; 5-fluorouracil, OHP; oxaliplatin, SN-38; irinotecan, nt; no treatment.

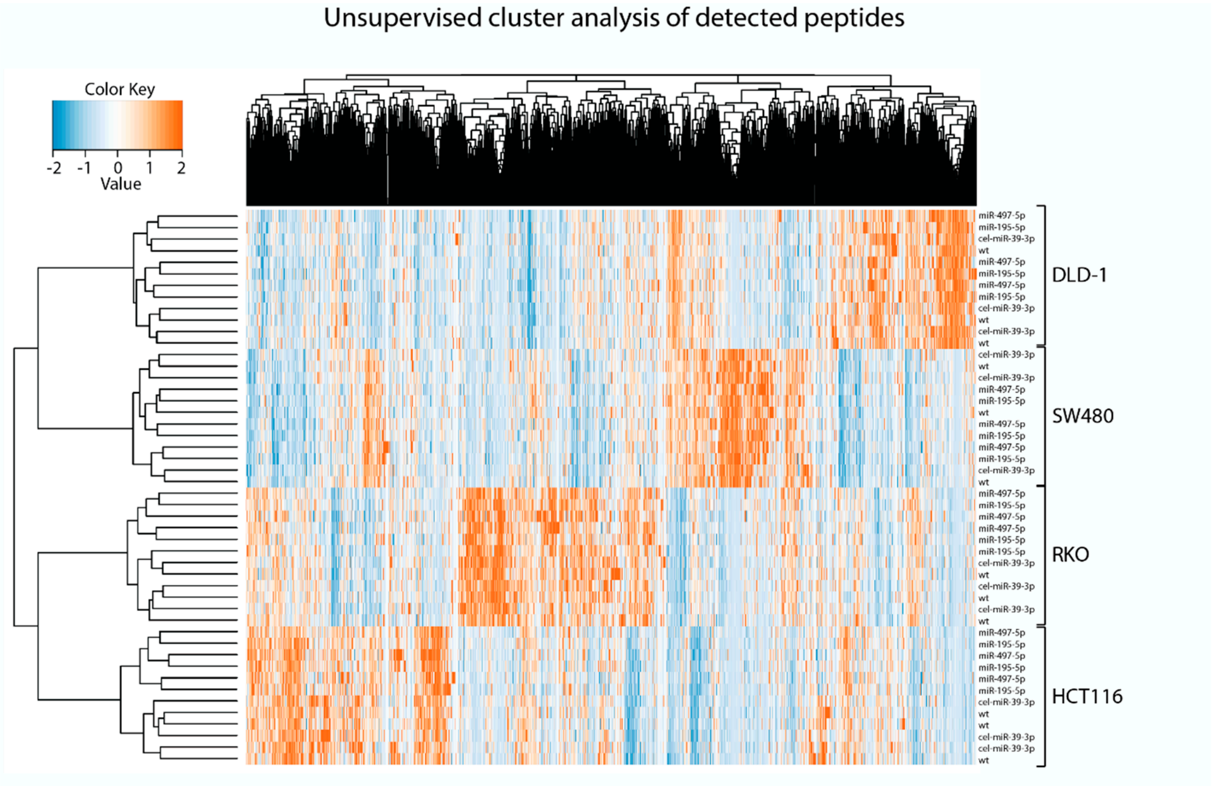

Supplementary Figure S3. Unsupervised cluster analysis of detected peptides in CRC cells. Each row represents detected peptides per condition (wild-type, cel-miR-39-3p, miR-195-5p mimic or miR497-5p mimic) per cell line (HCT116, RKO, SW480 or DLD-1). Each column represents a single peptide. Heatmapscore: z-score of normalized spectral count. wt: wild-type.

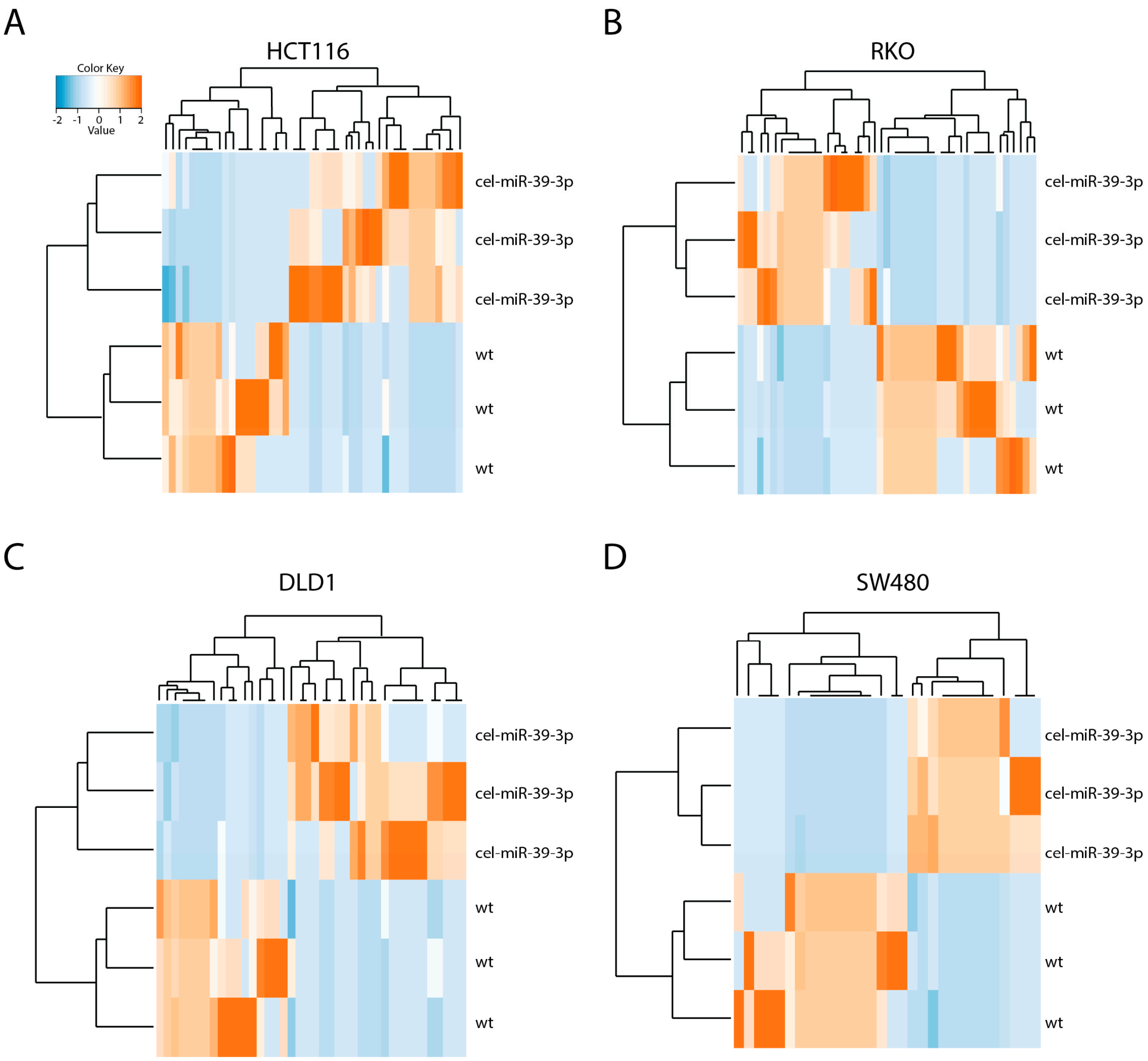

Supplementary Figure S4. Supervised cluster analysis of differentially expressed peptides between wild-type cells and negative control transfected cells. Significant different peptides in supervised clusters for HCT116 (**A**), RKO (**B**), DLD1 (**C**) and SW480 (**D**): p<0.05 not FDR corrected. Rows represent independent proteomic analyzes for each condition, cel-miR-39-3p and wt, performed in triplicate. Columns represent differentially expressed peptides. Heatmapscore: z-score of normalized spectral count. wt: wild-type.

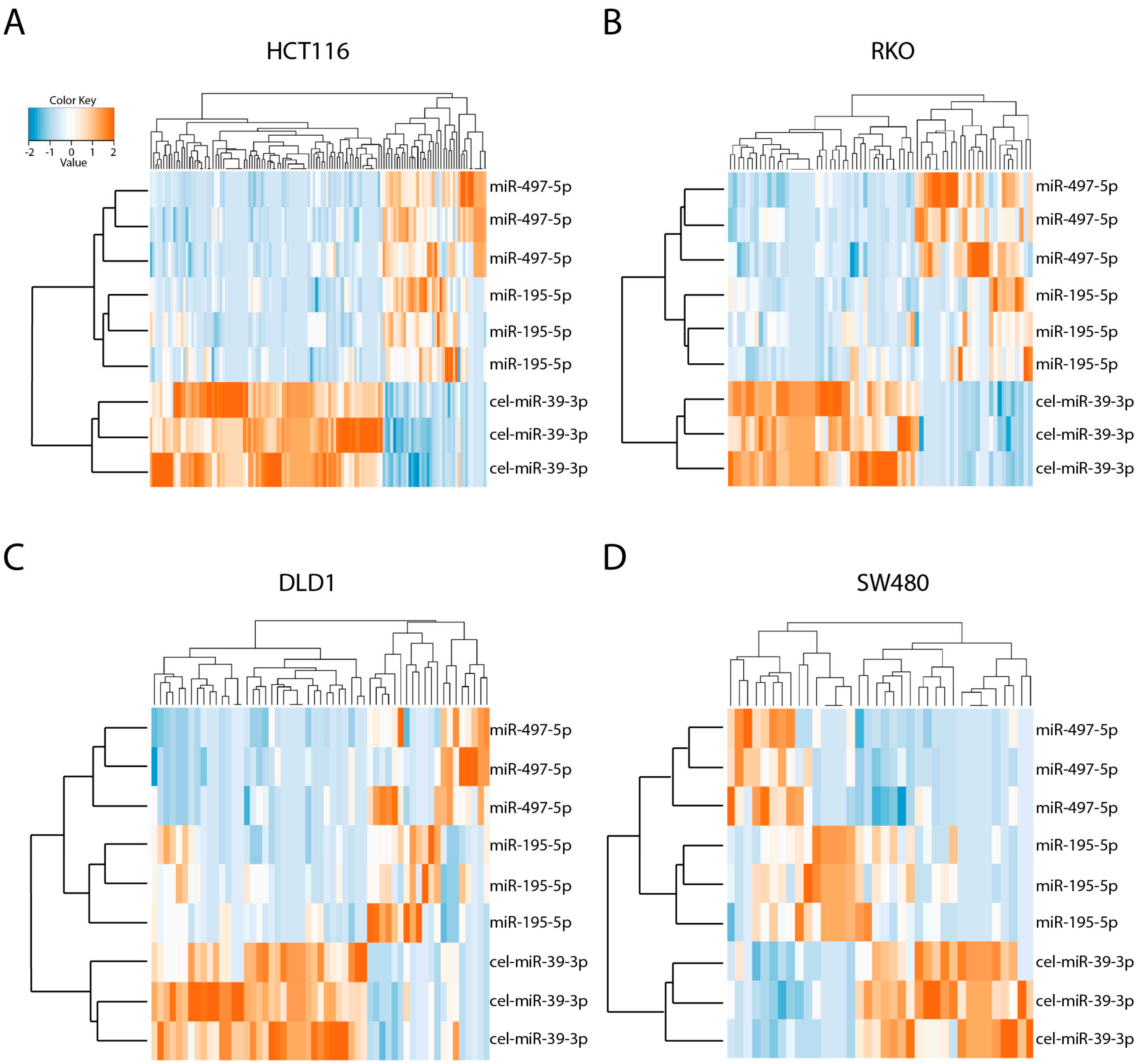

Supplementary Figure S5. Supervised cluster analysis of differentially expressed peptides between negative control transfected cells and combined miR-195-5p and miR-497-5p transfected cells. Significant different peptides after transfection with miR-195-5p and miR-497-5p in supervised clusters for HCT116 (**A**), RKO (**B**), DLD1 (**C**) and SW480 (**D**): $p<0.05$ not FDR corrected. Rows represent independent proteomic analyses for each condition, cel-miR-39-3p, miR-195-5p and miR-497-5p, performed in triplicate. Columns represent differentially expressed peptides. Heatmapscore: z-score of normalized spectral count. wt: wild-type.

Supplementary Table S1. Characteristics of the CRC cell lines included in this study. MSI: microsatellite instable, MSS: microsatellite stable, CIMP: CpG island methylator phenotype, CIN: chromosomal instability, wt: wild-type, mut: mutated. Characteristics were adapted from Ahmed *et al.* (1) CDKN2A mutation status was adapted from the COSMIC library (Catalogue of Somatic Mutations in Cancer), http://cancer.sanger.ac.uk/cosmic.

Cell line	MSI status	CIMP panel 1	CIMP panel 2	CIN	KRAS	BRAF	PIK3CA	PTEN	TP53	CDKN2A
RKO	MSI	+	+	-	wt	V600E	H1074R	wt	wt	wt
HCT116	MSI	+	+	-	G13D	wt	H1074R	wt	wt	mut
DLD-1	MSI	+	+	+	G13D	wt	E545K;D549N	wt	S241F	wt
SW480	MSS	+	+	-	G12V	wt	wt	wt	R273H;P309S	wt

Supplementary Table S2: Significant up- and downregulated peptides after transfection of CRC cells with miR-195-5p mimic and miR-497-5p mimic. Colors represent coinciding up- or downregulated peptides in at least two cell lines per mimic; red (down miR-195-5p), orange (up miR-195-5p), green (down miR-497-5p), blue (up miR-497-5p). Cut-off for significance was p<0.05 and FC<-3 or FC>3.

HCT116			
miR-195-5p		miR-497-5p	
Down	Up	Down	Up
TPX2	PPP3CA;PPP3CB	TPX2	PPP3CA;PPP3CB
TOP2A	DHRS7	TYMS	ZC3HAV1L
KIF11	FAM160B1	KIF23	GLIPR2
BUB1B	MVP	TOP2A	P4HA1
TYMS	RAB8B	CHAF1B	CAP2
TUT1	ZC3HAV1L	KIF11	FAM160B1
NLRP2	PDHX	TACC3	PDHX
CCNB1	CAP2	NMD3	PITRM1
NCAPD2	PITRM1	NCAPD2	DHRS7
PLK1	CTCF	NOP53	BDH2
USP3	P4HA1	WDHD1	CBX8
ANLN	PHLDB2	CCNB1	CDKN2AIP
BRD1	STXBP2	CBFB	DDHD1
ABCF2	CLIP1	POLR1A	GLRX
KIF4A:KIF4B:KIF6	ARMC6	AOR	STAMBP
KIF23	CBX8	PLK1	DERA
KIF2C	DDHD1	PRIM2	RAB8B
UBE2Z	NDUFC2:NDUFC2-KCTD14	USP3	SEC63
TACC3	PDCD4	ANLN	ARHGAP1
IRF2BP2	PTPN6	ATL2:ATL1	ZNF512
WASL	TNKS1BP1	CCNT1	APOE
ZNF622	UBE2D2	HELLS	CASP3
HECTD1	BDH2	ERCC6L	DSCR3
SLC12A2	GLRX	ABCF2	GSN
MKLN1	RAB5B	KIF4A:KIF4B:KIF6	INPP5K
VPS4A	GTF3C1	CLUH:PCDHAC2	KMT2C
WDHD1	RBM7	DIAPH1	MVP
NMD3	RNASET2	UBE2Z	NXN
TIMELESS	DLG1:DLG2	IRF2BP2	RAB5B
BCAS2	NOL10	SMNDC1	TOP2B
CTNNB1		ZNF622	UFSP2
ABCF3		SLC12A2	STXBP2
CDC20		MKLN1	PPCS
CDCA3		POMP	ATPAF2
RACGAP1		VPS4A	MPI
CHUK		PRIM1	NIT1
CREBBP		NLRP2	SET
KEAP1		BUB1B	EOGT
PHIP		RACGAP1	DNAJB6:DNAJB3:DNAJB8:DNAJB2:DNAJB7
RNF138		MOV10	PPP1R10
RPS6KA3:RPS6KA6:MAST3:MAST2:MAST4		ABCF3	PIP4K2A
SPTY2D1		CHUK	ADK
TOE1		KEAP1	FAH
UPF2		MRPL19	DLG1:DLG2
ZWINT		TOE1	HAGH
CDK4:CDK15:CDK14:CDK18		UPF2	
RIC8A:RIC8B		ZWINT	
MSH2		CDC20	
NOP53		CDCA3	
ZMPSTE24		TMA16	
CHAF1B		CKS1B	
DDX51		RNF138	
RFC1		NOL11	
PALLD:MYPN		SPTY2D1	
GPATCH4		SUZ12	
ANKRD17		RPS6KA3:RPS6KA6:MAST3:MAST2:MAST4	
CKAP2		TXLNA	
CLASP2		GTF3C2	
CUL4A		WDR6	
GOSR1		MSH2	
HSF1		GAPVD1	
LUZP1		CDK4:CDK15:CDK14:CDK18	
MRPS28		WDR75	
MUT		GID8	
RRP36		GPATCH4	
SHCBP1		USP19	
TMEM70		ANKRD17	
TONSL		CKAP2	
UBE2G2		GOSR1	
ZNHIT6		HIGD1A	
PACSIN3		LUZP1	
ODPR		SHCBP1	
CETN2:CETN1		TONSL	
DNAJB14		PACSIN3	
SRPRA		SCAF4	
DCAF1		SEC24A	
EPB41		UBFD1	
PTPRF:PTPRD:PTPRS		CETN2:CETN1	
SMG9		DNAJB14	
		FASTKD2	
		SRPRA	
		ISY1	
		LAMC1	
		PTPRF:PTPRD:PTPRS	
		ELL	
		RP9	
		SMG9	

RKO			
miR-195-5p		miR-497-5p	
Down	Up	Down	Up
SMTN	HMOX1	SMTN	OCIAD2
LAMB3	OCIAD2	SRPRA	CAP2
TPX2	CDK7	TMUB1	ARHGAP18
ERCC6L	TMX3	KIF11	NEK9
AOR	WDR18	PBK	DNAJC13
TMUB1	UBA3	TYMS	TRMT1
OGDH:OGDHL	SETD7	LUZP1	CYR61
SPCS1	CDK5	CA2	ZNF512
ATAD2	C11orf54	EXOSC2	ACY1
NECAP1	ACY1	UGT8	PIK3C2A
SBNO1:SBNO2	CYBA	NRBP1	TTYH3
ANKRD17	GALK1	SPTBN2	ABCC4
LUZP1	PAAF1	LSG1	DAP3
KIF11	PPP2R5C	AOR	CPT2
LSG1	CAP2	PYCR3	HMOX1
GFM1	NEK9	ATAD2	NUCKS1
CBR3	SMARCD1	GTF3C3	PTBP3
WDHD1	TRMT1	MSI1	AKAP2
ANLN	USP47	SMURF2	CD46
KIF2C	DERL2	ANKRD17	DERL2
TACC3	PDHX	GET4	KBTBD8
ANKRD52	DAP3	TACC3	NDRG3
CHMP6	APRT	USP3	ORMDL3
DUSP11		ANLN	PAAF1
HAUS7		ANKRD52	PCCB
WDR76		KCTD5	TMEM159
ZC3H13		KIF2C	LSS
ZNF48		KIF3B	RAP2B:RAP2A
CHAC2		RBM6	ATP5J
ZFAND5		WDR76	SETD7
RIOX1		CHEK1	IKBIP
DCAF1		ZNF622	TERF2IP
NT5C3A		DNAJC5	TJP2
CHUK		EXOG	WDR18
CLASP1		NT5C3A	ARHGAP12
AKAP1		CHUK	INF2
MORF4L2		CLASP1	RASA3
TPM1		TPM1	USP47
		ERCC6L	ADAM9
		CDK17	ABCD1
		AK3	POBP1
			UBA3
			PRPF31

DLD1			
miR-195-5p		miR-497-5p	
Down	Up	Down	Up
ABCF3	A2M	TPX2	TJP3
VRK2	TMX3	KIF11	LARS2
ARHGAP5	AMPD2	ANLN	MPRIP
HMMR	CAVIN1	DNMT1	USP47
ANKRD17	ECE1	RUNX2	TNS4
SLC1A4	EXOSC3	ARHGAP5	CIAO1
USP15	NECAP2	HELLS	PHLDB2
CCNB2	PGM2L1	NMD3	ALB
CDCA3	TNS4	CPD	CAP2
TBC1D2	MPRIP	ERAL1	ABHD6
SPTY2D1	TAF7	FAM111B	COG6
ANLN	ZFYVE16	HMMR	DNASE2
KIF11	UFSP2	MRPS30	ECI1
SPCS1	IRGO	SCFD1	IRGO
TACC3	USP47	ANKRD17	LRBA
WDR76	ALB	SLC1A4	MTF2
ADGRG1		USP15	PGM2L1
ATP5I		WBP11	PLCG1
EXD1		NCAPH	GDI1
MAP4K3		PCLAF	A2M
NSD3		CDCA3	TMX3
PLBD2		CPSF2	AMPD2
RNF167		NUF2	MBLAC2
CHTF18		ABCF3	NDUFB4
DNAAF5		TK1	CHID1
GTF3C2		DCAF8	SCAF11
MASTL		WDR76	SH3PXD2B
RAD18		PLK1	PIP4K2A
COX6C		CKAP2	
PPP6R3		ALDH16A1	
ARFGAP2		BCLAF1	
UBE3A		EMC8	
PCF11		MRPL41	
MORF4L2		NSD3	
		PTCD1	
		PUS1	
		RNF167	
		SCAMP4	
		STX18	
		KIF23	
		UBE2C	
		DNAAF5	
		MASTL	
		PNISR	
		PPP6R3	
		MPZL1	
		ARFGAP2	

SW480			
miR-195-5p		miR-497-5p	
Down	Up	Down	Up
TACC3	PPP1R10	UBE2C	DOCK9
PLBD2	GNS	APP	TM9SF3
BCAS2	ABCD3	HELLS	ADK
SMG9	THOC3	ANLN	GPD1L
AGRN	GPD1L	HMMR	ACTR1B
ATAD3B	MLKL	BCAS2	GNS
CDK9	RSF1	FXN	HLA-A
DPP7	TMA16	TPRG1L	NIPBL
FNBP4	VPS16	FAM111B	STAM2
FXN	FUCA2	MAP7	PPA2
MAP7	MOCS3	PLK1	PPP1R10
MRPS18A	OSGEP	APOE	GLB1
NDUFA9	XPNPEP1	ATAD3B	MXRA7
NOB1	CEP78	DHRS2	ARF6
PFDN4		FAM207A	ARHGEF7:ARHGEF6
POLD1		NSA2	ECI1
PRPH		PFDN4	ARMC6
SEC31A		POLR1D	CAMSAP3
ABCF3		RPA3	XPNPEP1
COX6C		RPP40	PGM3
AK3		SLC5A6	
SNRPB2		TAF10	
CBR3		TACC3	
		ERCC6L	
		BCS1L	
		HIST1H3A:HIST3H3:H3F3C	
		UTP3	
		FOXK1	
		ITGA2	
		MAP2K3	
		CSNK1A1:CSNK1A1L	
		SETD3	
		ABRAXAS2	
		ELOA	
		POLRMT	
		RFC1	
		SMG9	
		TRMT1	
		CETN2:CETN1	

Supplementary references

1. Ahmed D, Eide PW, Eilertsen IA, Danielsen SA, Eknaes M, Hektoen M, et al. Epigenetic and genetic features of 24 colon cancer cell lines. Oncogenesis. 2013;2:e71.
